# Patient involvement in clinical trials: a paradigm shift in research

**DOI:** 10.1186/s13023-025-03573-y

**Published:** 2025-02-10

**Authors:** Jordi Pijuan, Francesc Palau

**Affiliations:** 1 Neurogenetics & Molecular Medicine Research Group, Institut de Recerca Sant Joan de Déu and CIBERER, Barcelona, Spain; 2 Neurogenetics & Molecular Medicine Research Group, Institut de Recerca Sant Joan de Déu and CIBERER, Hospital Sant Joan de Déu, Barcelona, Spain

Clinical research plays a pivotal role in studying health and disease, driving the acquisition of new medical knowledge and enhancing patient care. Over the past decade, the landscape of clinical trials has expanded significantly on a global scale. These trials are essential for evaluating new drugs, treatments, and interventions on human subjects to determine their safety, effectiveness, potential side effects and overall impact on patient well-being. Patients with rare and ultra-rare diseases are particularly important within this framework, as their involvement in clinical trials is essential for elucidating underexplored pathologies and fostering the development of novel therapies. Despite this importance, many clinical trials encounter substantial hurdles that hinder their success. Common issues include inadequate participant recruitment, which may lead to non-representative samples, poorly crafted study designs that fail to address patient needs, low rates of trial completion, and ineffective dissemination of research findings. These challenges can significantly impede the advancement of clinical research and ultimately affect patient outcomes negatively [1]. Recent studies indicate that patient involvement in various aspects of research remains suboptimal, particularly during the initial stages of study design and objective formulation. This issue is especially critical in the context of rare diseases, where the limited patient population highlights the necessity for designing highly personalized studies and maintaining continuous engagement with patients and their families. Furthermore, there is often insufficient engagement with patients regarding feedback and experiences throughout the research process. Such input is essential for enhancing patient safety and mitigating adverse events [2]. Consequently, there is an urgent need for patient and public involvement and engagement (PPIE), which plays a significant and impactful role in conducting patient-centered clinical research [3]. Integrating PPIE, along with caregivers and service users, can yield valuable insights into patient experiences, bolster participant recruitment, and enhance the developmental phases of clinical trials. These contributions ultimately foster improved health outcomes and benefits for all parties involved. By incorporating PPIE, researchers are better equipped to align their inquiries with the genuine concerns of patients, resulting in significant enhancements to patients’ quality of life [4].

Historically, clinical research has predominantly centered on evaluations and analyses from a clinical standpoint, often sidelining the perspectives of patients. Recent trends, however, indicate a growing acknowledgment of the significance of patient perception in research. Patients are transitioning from being mere subjects to active participants in the research process. This paradigm shift is vital for fostering a more nuanced understanding of clinical inquiries. The United Kingdom’s National Institute for Health and Care Research (NIHR) has been at the forefront of advocating for the awareness, implementation, and assessment of PPIE [3]. In support of researchers, the NIHR has created several resources, including guidelines that function as practical frameworks for planning and evaluating PPIE strategies within research projects [5]. Recently, similar initiatives have emerged in other European countries, as well as in North America and Australia, to assist researchers in integrating patients and the public in all research aspects [2].

A prominent example illustrating effective collaboration among physicians, researchers, and patient organizations is the European Reference Network for Rare Neuromuscular Diseases (EURO-NMD) registry. This registry, which is structured to gather data from individuals afflicted with neuromuscular diseases, addresses the unmet needs of patients by actively involving them at every stage. Patients contribute medical-grade, patient-originated data through patient-reported outcomes, which are invaluable for long-term disease monitoring, treatment evaluation, and the foundational design and development of clinical trials [6].

The engagement of patients in clinical research initiatives is crucial for enhancing the relevance and effectiveness of studies aimed at improving health outcomes. This involvement can be articulated through five fundamental dimensions: (*i*) *Expertise*: patient organizations possess significant experience and insights related to the specific needs and preferences of the patient community. This knowledge is vital for aligning research objectives with actual patient concerns. (*ii*) *Patient involvement*: actively engaging patients at every stage of the research process fosters trust, encourages higher participation rates, and instills a sense of ownership and collaboration among stakeholders. (*iii*) *Resource leveraging*: patient organizations play a critical role in optimizing available resources, thereby maximizing the impact of funding contributions. They do this by providing staff time, specialized knowledge, and various other resources that enhance research budgets and extend the overall scope and effectiveness of projects [7]. (*iv*) *Collaboration*: establishing a registry facilitates robust partnerships among patients, researchers, and other key stakeholders. This collaboration ensures a coordinated effort toward achieving shared research goals. (*v*) *Co-governance*: patient organizations are recognized as equal partners in the research process, holding the same rights and responsibilities as other collaborators, thereby integrating patient perspectives and priorities at every research phase from initial design to final results dissemination [3]. The transformative potential of meaningful patient engagement in clinical research is exemplified through initiatives such as the EURO-NMD registry. By harnessing patient expertise and advocating for active collaboration, such initiatives significantly enhance the quality, relevance, and impact of research endeavors, ultimately benefiting all parties involved. Nonetheless, the routine integration of patients in every phase of clinical research remains inconsistent across various studies. Prioritizing the voices of patients and caregivers, while systematically gathering relevant information, directly contributes to improving the quality of life for those affected. This approach informs the research process by assisting in the development of studies (from project conception through their design and eventual evaluation). Moreover, it enhances treatment follow-up protocols, facilitates sound medical recommendations, and supports informed decision-making in medication development. By promoting methodological advancements and ensuring equitable access to new therapies, these collaborative efforts ultimately lead to improved clinical trial outcomes, making a significant impact on patient care.

The promotion of greater responsibility among researchers, clinicians, funders, and publishers is crucial for advancing PPIE. There is a pressing need for enhanced research and outreach initiatives to elevate societal awareness of these matters transparently. It is particularly vital to focus on the needs of patients with rare and ultra-rare diseases, whose voices and requirements are often underrepresented. Establishing universal guidelines will help ensure that all stakeholders (patients, caregivers and health professionals) can engage meaningfully in the developmental processes of healthcare initiatives. Given that health and disease affect us all, fostering collective involvement is essential for driving progress in this field.
